# Flash, the emperor and policies without evidence: counter-terrorism measures destined for failure and societally divisive

**DOI:** 10.1192/pb.bp.116.053603

**Published:** 2016-04

**Authors:** Kamaldeep Bhui

**Affiliations:** 1 Queen Mary University of London

## Abstract

Governments around the world are uniting in trying to defeat terrorist movements. In this context, recent counter terrorism laws in the UK place public duties on all citizens to help prevent terrorism. Yet, the science of predicting rare events such as terrorist offending yields consistently poor results. There are ethical, clinical and scientific dilemmas facing the professions if we are to investigate social, religious and political belief systems in routine assessment in order to inform judgements about terrorist offending risk. A balanced and evidence-based approach is necessary.

Radicalisation, the process by which ordinary individuals come to sympathise with and support violent protests and terrorism, is thought to include both social and psychological determinants and vulnerabilities that shape otherwise healthy young people to engage with and adopt terrorist ideology.^1^ Actual terrorist offending is rare and has challenged a wide array of experts from a variety of different disciplines including historians, scientists, forensic, health and social care professionals, and stakeholders from the criminal justice agencies. Preventing radicalisation and terrorism is even less well understood, and has a smaller evidence base than homicide. There is insufficient research evidence to propose any single model or mechanism by which radicalisation leads to terrorism, and then even less that is consistent about the role of mental illness, although more is emerging about the role of emotional and psychological factors.^2^ Most knowledge about terrorism comes from reconstructed biographies of convicted terrorists where pathways are sought from ordinary citizen to a phase of pre-radicalisation, followed by indoctrinated commitment to terrorist causes.^2,3^ These retrospective accounts cannot be verified objectively and are subject to recall bias but adopt the only approach known in criminological investigations. Further arrest and conviction necessarily lead the individual to re-envisioning their identity and sense of belonging. Their own narrative of who they are and what has happened is shaped by the need to justify their actions when asked to explain how they came to commit offences labelled as terrorism.^2,3^

Little research has explored the early phase of radicalisation in the UK and other high-income countries, when individuals turn on their countries and give up friendships, family, freedom and opportunity available to them. Even less research has explored how ordinary people living in the community, ostensibly gaining from and giving to their society, decide to attack their community and country.^4^

Terrorism seeks to secure political objectives through violence, fear and intimidation of both populations and politicians. Consequently, governments are obliged to respond by attempting to secure the safety of their citizens as well as entering into international negotiations on foreign policy, security and counter-terrorism strategies. Although terrorism has a long history, the recent discourse foregrounds people of Muslim heritage in high-income countries such as the UK, the USA, Canada and Australia. More recently, France and Belgium have been drawn into this concern.^5^ It is known that the vast majority of terrorist offences take place in countries with a Muslim majority and low levels of income, and indeed the victims are mostly of Muslim heritage, although the evidence for terrorism being linked to Muslim countries is not universal as many such countries enjoy peace and prosperity.^6^

The immediacy of terrorist threats, often unexpected and in spite of significant counter-terrorism intelligence and investment, has provoked a crisis in confidence and strategy, leading to calls for urgent intervention locally and internationally. Within this counter-terrorism discourse, the place of religious ideology is conflated with orthodox religious beliefs, and the political basis of terrorist acts disguised as religious rhetoric is overlooked. Attacking terrorism through a religious idiom is not soundly based on evidence. Not all Muslims are at risk of terrorism and many Muslim countries do not experience terrorism. Extremist political interpretations are a minority but are reacted to as if these are mainstream religious beliefs. If clinicians are asked to make judgements about terrorism risk, radicalisation or even cultural variations of religious practices and whether these fall within norms, then community advocacy and partnership is required to help make those judgements. Clinicians are also expected to ask about and problematise the nature of religious beliefs and the boundaries with political beliefs. This very topic has been contentious. On the one hand, professional secular boundaries are necessary to protect the patient and clinician in areas of ethical controversy,^7^ but a culturally sensitive and competent enquiry is necessary to discern delusional beliefs, as distinct from culturally acceptable beliefs and religious practices.

In part the strategy of terrorism is to provoke a Draconian, oppressive counter-response in order to exonerate perpetrators and vilify governments of Western democracies, which then risk an unwanted by-product. If policies target Muslim or religious populations, it demonstrates to people of Muslim heritage, or strong religious affiliations, that they are not valued equally to other citizens; indeed, their role in resolution and protection of their society is not recognised or exploited to promote cohesion and safer societies. Religiosity itself becomes a source of suspicion and concern. Understanding the construction of religious experience and the psychological costs of holding religious beliefs (perhaps with contradictory and contested evidence, hence the need for faith and belief) is the subject of much cultural, philosophical and neuroscientific research.^8^ We need to know far more in order to separate beliefs that are benignly religious from those that include political motivations and incite violence but are disguised through religious rhetoric; without this knowledge clinicians would face an onerous and unscientific set of expectations. Regrettably, the current UK government's counter-terrorism responses, specifically the Prevent programme, have been criticised for begetting exactly this unintended consequence.

The most recent counter-terrorism Bill seeks to invoke a public duty on all citizens and public servants of identifying a potential terrorist threat as early as possible. The implications for healthcare and educational institutions and other employing organisations are that they should have a responsibility to carry a high index of suspicion. Further, they are obliged to intervene when they come across seemingly suspicious individuals or groups who might be harbouring terrorist intentions. Although well-intentioned, this proposal has been met with a rather more concrete interpretation by some in public institutions. For example, in education, even in primary schools, enthusiastic early adopters misclassified individuals as being a potential terrorist threat without fully appreciating the lack of any valid method of prediction.

In mental health services, there exists a special concern that people with poor psychological health and psychiatric difficulties are particularly vulnerable to exploitation and persuasion, especially if they are additionally distressed and isolated and should come into contact with nefarious, infectious terrorist ideology. Emerging information suggests that those who commit terrorist offences rarely have severe mental illnesses, specifically disorders with symptoms of hallucinations and delusions. Nevertheless, it is sometimes difficult to disentangle political ideology and commitment from delusional or overvalued ideas, when these are held by a peer group from a similar cultural background, even if a minority.^8,9^ In rare situations it has been found that so-called ‘lone wolves’ acting in isolation from persuasive terrorist organisations appear to be at high risk of having mental health problems and acting erratically and perhaps impulsively, to seek redress for perceived insult or assault on their cultural religious beliefs, assuming the terrorist ideology to be true.^10^ In addition, vulnerable individuals seek potent self-identity and influence through joining gangs or shared interest groups, perhaps not realising the gravity of potential offending in which they may be later involved. Forensic psychiatrists and psychologists of course have to debate these issues daily. But terrorism is a form of offending given special status and investment as the new evil that must be combated. It is with this zeal that some interpret their public duties.

In mental healthcare we are experienced in managing risks of suicide, self-harm, violence and homicide. Accepting that the science of prediction of rare events is limited, it is necessary to follow established safeguarding processes and procedures in an effort to minimise the potential for unwanted outcomes.

The UK government's counter-terrorism provisions could be understood in this context: they are perhaps simply asking us to ensure we maintain a high index of suspicion, optimal safeguarding, and most importantly, do not consider concerns about potential terrorist offending to be outside the remit of our public duties as citizens. The implementation of such activities may be difficult to marry up with the responsibilities of a healthcare professional or indeed any other public servant, as it requires more resources and time, as well as discussion and documentation. It also risks stigma and the alienation of people seeking help from any official service or channel.

I have some sympathy with Derek Summerfield's position^11^ in that medical ethics mandate confidentiality and the protection of an individual's medical information and health, although clearly this has to be balanced with considerations of risk to others. Yet the implication that health professionals are somehow to routinely seek out any index of potential terrorism overstates the scientific knowledge about who is a terrorist offender, and about what radicalisation is as a process and who might be vulnerable to it.

Further deficiencies in scientific knowledge fail to help us understand how radical ideas can exist as extremist political ideology or philosophy, and how political ideology seeks to exploit religious rhetoric, as if appealing to all people of Muslim heritage. In our studies of sympathies for violent protest and terrorism among South Asian populations of Muslim heritage, ordinary citizens living in the community, mostly employed and educated, we found the stereotypical characteristics such as poverty disadvantage and discriminatory experiences as unimpressive correlates of pre-radicalisation sympathies.^12,13^ Migrants in fact were less likely to hold such views as were those with poor health and living in areas of low social capital. Similarly, work undertaken by specialist researchers working for governments and independent researchers has not identified a range of predictive variables, reinforcing that the only approach available is one of safeguarding, careful risk assessment and management. It is known that patient and public involvement improve the quality of public health and societal research, especially in the realm of preventive science, so more active involvement of communities is needed. Although research on those at risk of offending or convicted terrorists is necessary, considerable care needs to be exercised with regard to ethics and safety of researchers and the public, as well as to not undermine the efforts of criminal justice agencies.

In part the appeal of the terrorist threat is an infectious but noxious idea with which to grapple, reflecting the human fascination with transformation from hero to villain, as exemplified in popular film, children's cartoons, and theatre. Woody and Buzz Lightyear in the film *Toy Story*, Flash Gordon fighting an emperor, Luke Skywalker in Star Wars, and Harry Potter all struggle with their identity as villain or hero. All battle malevolent forces while being changed by them, and yet surviving, overcoming and defeating the appeal of violence and evil which is portrayed as pleasurable. We must ensure our counter-terrorism response and public citizen duties do not engage with the realms of fantasy. They must be subject to intense, intelligent, evidence-based efforts to safeguard our patients wherever possible, while at the same time promoting mental health and well-being even in treacherous times of conflict and, for some, financial ruin and disconnection. All should prioritise safeguarding, while doing away with policies without evidence.
